# A Narrative Review of School-Based Physical Activity for Enhancing Cognition and Learning: The Importance of Relevancy and Integration

**DOI:** 10.3389/fpsyg.2018.02079

**Published:** 2018-11-02

**Authors:** Myrto Foteini Mavilidi, Margina Ruiter, Mirko Schmidt, Anthony D. Okely, Sofie Loyens, Paul Chandler, Fred Paas

**Affiliations:** ^1^Priority Research Centre for Physical Activity and Nutrition, University of Newcastle, Newcastle, NSW, Australia; ^2^Early Start Research Institute, University of Wollongong, Wollongong, NSW, Australia; ^3^Department of Psychology, Education and Child Studies, Erasmus University Rotterdam, Rotterdam, Netherlands; ^4^Institute of Sport Science, Universität Bern, Bern, Switzerland; ^5^University College Roosevelt, Utrecht University, Middelburg, Netherlands

**Keywords:** physical activity, embodied cognition, movements, learning, children

## Abstract

Engaging in regular physical activity can have substantial cognitive and academic benefits for children, and is generally promoted for its beneficial effects on children’s physical and mental health. Although embodied cognition research has convincingly shown the integral relationship of the human body and mind, in schools physical activity and cognitive activity are typically treated as unrelated processes. Consequently, most physical activities used are neither sufficiently relevant for nor fully integrated into the learning tasks. In reviewing the literature regarding the integration of physical activity into education to promote cognition and learning, two main lines of research emerged: exercise and cognition research vs. embodied cognition research. In this narrative review, we describe these two separately evolved schools of thought, highlighting their differences and commonalities. In categorising the existing studies on a 2 × 2 matrix, concerning the two main categories of relevance for and integration into the learning task, it becomes clear where the different foci lie, and how both lines of research could profit from learning from each other. Finally, a new instructional model that integrates task-relevant physical activities into the cognitive/learning task is proposed to inform both further research and educational practice.

Human movement, cognition, and learning are inextricably bound. Starting in early life, children act upon and understand the environment using mainly sensorimotor actions (Thelen et al., 2001). Broad changes in perception, cognition, and behaviour appear with the development of a child’s sensorimotor repertoire (Piaget, 1970). Different types of motor experiences are prevalent throughout the development from childhood into adulthood, such as reaching and grasping movements (Daum et al., 2009), gross motor patterns of varying complexity mastered in the context of physical activity and sport (Cross et al., 2006), and gesturing (Goldin-Meadow and Beilock, 2010). All these forms of human movements have been shown to affect cognition and learning.

In searching for underlying mechanisms explaining the connexion between physical activity, cognition, and learning, two main explanations deriving from two completely different lines of research can be found in the literature: physiological and cognitive. On the one hand, exercise and cognition research has predominantly referred to the physiological changes induced by single bouts (e.g., enhanced catecholamine levels) or multiple bouts of physical activity (e.g., changed brain structures) as an explanation for the inter-relatedness of physical activity and cognitive functioning (e.g., Etnier et al., 1997; Donnelly et al., 2016). On the other hand, embodied cognition research has mainly focused on cognitive explanations, discussing them in the context of more subtle movements, such as gestures, and more recently whole-body movements (e.g., Lindgren et al., 2016), influencing cognitive processes and learning (e.g., Goldin-Meadow and Beilock, 2010).

The main goal of this narrative review is to describe both research traditions (i.e., exercise and cognition research and embodied cognition research), and to review the literature to determine whether combining these two approaches may provide a synergistic benefit for both research and educational practice. The included literature will be described in terms of whether the movements are relevant and/or integrated with the cognitive/learning task. In doing so, one can identify that the first research tradition focuses on physiological explanations for the general health and cognitive benefits of gross motor movements in the form of physical activity or exercise, but without considering the relevance of the movements for the learning task. Typically, gross movements are not integrated (i.e., no temporal connexion of the movements with the learning task) with academic lessons, and are not meaningful or congruent with the task. The second research tradition focuses on cognitive explanations of subtle/fine or whole-body movements that are relevant to the learning task. Researchers in this tradition, however, do not consider the physiological benefits of physical activity or exercise.

In describing the two lines of research, the concepts of physical activity, exercise, and physical fitness are based on the definitions provided by the Centres for Disease Control and Prevention (2017):

1. Physical activity: “any bodily movement produced by the contraction of skeletal muscle that increases energy expenditure above a basal level.”
2. Exercise: “a subcategory of physical activity that is planned, structured, repetitive, and purposive in the sense that the improvement or maintenance of one or more components of physical fitness is the objective. Exercise and exercise training are frequently used interchangeably and generally refer to physical activity performed during leisure time with the primary purpose of improving or maintaining physical fitness, physical performance, or health.”
3. Physical fitness: “the ability to carry out daily tasks with vigour and alertness, without undue fatigue, and with ample energy to enjoy leisure-time pursuits and respond to emergencies. Physical fitness includes a number of components consisting of cardiorespiratory endurance (aerobic power), skeletal muscle endurance, skeletal muscle strength, skeletal muscle power, flexibility, balance, speed of movement, reaction time, and body composition” (Centres for Disease Control and Prevention, 2017).

Acknowledging the aforementioned definitions, the terms “physical activity” and “movement” can be used interchangeably. However, we will use the term “physical activity” to label those activities which have been investigated in the exercise and cognition research tradition: mainly gross motor movements, which largely increase energy expenditure above a basal level. The term “movement,” on the other hand, will be used to describe activities which have been investigated in the embodied cognition research tradition: mainly fine motor movements, such as gestures, which only marginally increase energy expenditure.

Finally, we propose the third approach, which combines both traditional approaches by looking at integrated, task-relevant physical activities. Blending the physiological and cognitive research traditions to improve both cognition and learning can potentially provide the field of education with valuable insights that can be used to formulate more concrete guidelines for the effective integration of movements in learning environments.

## Exercise and Cognition Research

The exercise and cognition research tradition has its roots in ageing research (Colcombe and Kramer, 2003; Gajewski and Falkenstein, 2016). Interested in understanding how physical exercise could reduce the age-related decline in cognitive functioning, exercise scientists adopting a more medical perspective have tried to find the right dose of exercise to reach the largest cognitive benefits (e.g., Chang et al., 2012). Only in the last decades, there has been a shift toward the younger population, and concomitantly, interest in the effects of physical activity on children’s and adolescents’ cognition and brain development (Khan and Hillman, 2014; Pesce and Ben-Soussan, 2016). The historical foundation in ageing research coupled with the medical perspective may explain why this line of research is focusing on cognitive functioning as the main outcome variable, rather than on learning, and why the given explanations are so physiological in nature.

### Physiological Explanations for the Effects of Physical Activity on Cognition

The positive effects of physical activity are widespread across various domains of human life. The benefits of physical activity have been expanded beyond cardiovascular health and obesity prevention, and include improved cognitive functioning, as well as brain structure and activity (Khan and Hillman, 2014). Several findings indicated that improving cardiovascular fitness through regular exercise induced morphological changes to the brain and enhanced cognitive functioning in ageing humans (Kramer et al., 1999; Colcombe et al., 2004). Especially, executive functions (EF) appeared to be more susceptible than other cognitive processes to aerobic exercise (Colcombe and Kramer, 2003). EF is the part of cognition that encompasses effortful and goal-oriented functions, including inhibition, working memory, and cognitive flexibility (Baddeley, 1996; Miyake et al., 2000). These foundational components, form the basis for higher order EFs, such as reasoning, problem solving, and planning (Collins and Koechlin, 2012; Lunt et al., 2012). The working memory system is especially influential for memory, perception, and attention (Baddeley, 1992). It is comprised of the central executive and two peripheral systems, the visuospatial sketch pad and phonological loop. The central executive is responsible for the attentional control of behaviour, such as processing, storage, and coordination of information coming from the peripheral systems. The phonological loop holds acoustic or speech-based information, and is important for speech perception and production, whereas the visuospatial sketch pad is linked with visual perception and action.

Findings in older adults resulted in the EF hypothesis, proposing that exercise affect EF’s by inducing vascularisation, neurogenesis, and by altering synaptic processes in neural networks supporting EF, therefore influencing higher order thinking processes (Rasberry et al., 2011; Khan and Hillman, 2014; Verburgh et al., 2014). Children’s cognitive and neural development, and in particular EF and the supporting brain structures, may also be ameliorated by physical exercise (Diamond, 2000; Hillman et al., 2008; Diamond and Lee, 2011). Higher fitness levels correlate to greater school performance due to physiological alterations in the brain structure, e.g., larger hippocampal volume, neurogenesis, synaptic plasticity, oxygenation, and brain circuit of hormones and neurotransmitters among higher fit preadolescent children of 7–10 years old (Chaddock et al., 2010; Khan and Hillman, 2014; Chaddock-Heyman et al., 2016). Although 95% of the brain size is reached by age 6, grey matter volume in the frontal, parietal, and temporal lobes peak during 10–12 and 16–17 years, respectively (Giedd et al., 1999; Lenroot and Giedd, 2006; Khan and Hillman, 2014). Adolescents experience functional changes in EF, where increased activity has been observed in the prefrontal regions during the performance of social cognitive tasks (Blakemore, 2008).

Developmental neuroimaging studies have shown a gradually maturing sensorimotor system before the emergence of higher order EF, while neuroscientists have linked developmental changes in the brain with behavioural performance measures (e.g., memory function, task performance; Casey et al., 2005). Specifically, regions responsible for primary functions such as motor and sensory systems mature earlier than regions related to higher order association. Because the latter regions integrate these primary functions, the consensus is that EF is crucial for mental and physical health, academic success, cognitive, social, and psychological development (Diamond, 2013). It is considered even “more important for school readiness than intelligence quotient” (Bull et al., 2008; Diamond and Lee, 2011, p. 959), and positively affects math and reading performance throughout education (Blair and Razza, 2007). EF is subsequently correlated with on-task behaviour, aiding self-regulation, behavioural inhibition, and the ability to focus on classroom material despite internal or external distractions, which is essential for successful learning. Children show increased on-task behaviour after physical activity programmes at school (Riley et al., 2016), confirming that physical activity can positively affect classroom behaviour.

Until now, several meta-reviews in children report a favourable relationship between physical activity and aerobic fitness on the one hand, and cognition and brain function on the other hand. In 1997, Etnier and colleagues examined 200 studies, in which 134 were included. They indicated that acute exercise has a significant small positive effect (Hedge’s *g* = 0.36) on cognitive performance with children (6–13 years). Also, later in their meta-analysis with the same age group, Sibley and Etnier (2003), after having examined 118 studies and analyzed 44 studies, reported a similar overall effect size (Hedge’s *g* = 0.32). More recently, Donnelly et al. (2016) systematically reviewed the relationship between physical activity, fitness, cognition, and academic achievement and concluded that most research findings support the view that physical fitness, single bouts of exercise, and participation in physical activity programmes are beneficial for children’s cognitive functioning. Likewise, overall results of a recent narrative review and meta-analysis of Vazou et al. (2016) showed how chronic physical activity interventions positively impacted children’s cognitive functioning (Hedge’s *g* = 0.46). Finally, the meta-analysis of Verburgh et al. (2014) looked specifically at the relationship between physical exercise and EF in preadolescent children, adolescents and young adults. Overall, positive effects of acute physical exercise were found for both general (Cohen’s *d* = 0.52) and domain-related EF (i.e., inhibition and interference control, Cohen’s *d* = 0.46; working memory, Cohen’s *d* = 0.05). Although no effects of chronic exercise were found on general EF, positive effects were found for planning (Cohen’s *d* = 0.16).

### Type and Duration

Broadly speaking, there are two different methodological approaches to investigate the relationship between physical activity and cognitive functioning, which has consequently resulted in two distinct lines of research. The first type investigates the effects of chronic exercise (i.e., repeated bouts of exercise such as aerobic training; Tomporowski et al., 2015). The main goal of the habitual aerobic exercise programme is to enhance children’s cardio-respiratory fitness (Tomporowski et al., 2015). This improvement, in turn, may induce indirect yet more enduring effects to cognitive functioning, which are assessed as after the chronic aerobic exercise programme has finished (Tomporowski et al., 2008). The second line of research investigates the instant changes in cognitive functioning directly after acute bouts of aerobic activity (Tomporowski, 2003). Participants’ accuracy, response time, and speed on cognitive tests are assessed immediately after intense aerobic training.

For instance, single bouts of physical activity may provoke physiological arousal, facilitating the available attentional resources and engagement of cognitive functioning (Best, 2012). Chronic aerobic exercise generates functional morphological changes in the brain structures (i.e., larger amplitude and shorter latency of the P3 component in the frontoparietal network) critical to learning and memory, whereas acute exercise improves cognitive performance by activating neurochemical responses (Best, 2010; Brisswalter et al., 2002; Chang et al., 2012; Hillman et al., 2003; Tomporowski, 2003). Physical activity can enhance cognitive processes related to faster cognitive processing, and increase the allocation of attentional resources during encoding (Hillman et al., 2003). Erickson et al.’s (2015) review supports this view, concluding that greater physical activity and higher fitness levels in children is associated with larger grey matter volume in the hippocampus and basal ganglia, greater white matter integrity, and increased neural efficiency (e.g., improved functional connectivity) in adolescents and older adults. Nevertheless, grey matter reductions may also occur after late adolescence due to maturation, continuing to enhance EF as neural patterns respond to an input-dependent manner based on the environmental influences. Here, undesirable behaviors are pruned away in favor of desirable behaviors (Blakemore, 2008). For example, instead of just thoughtlessly initiating learning, students plan what and how they will learn beforehand, continuously monitoring their learning processes (e.g., metacognition; Flavell, 1979; Zimmerman, 2002).

Despite the positive associations among aerobic exercise, fitness, cognition, and academic achievement, there are many quantitative (i.e., type, amount, frequency, and timing) and qualitative aspects (i.e., task complexity, novelty, and diversity/variety, emotional activation, and selection of strategies) of physical activity in relation to cognition that remain to be explored. Here, exercise and cognition research has only recently began to shift the focus from the quantitative to the qualitative exercise characteristics (Pesce, 2012). Based on the simple idea that various physical activities may not only differ in their intensity, duration, and frequency, but also, for example, in their coordinative and cognitive complexity, this new perspective is almost exclusively restricted to children and adolescent samples in previous research (Vazou et al., 2016). Exercise psychologists working in this area of research are stressing the importance of the cognitive demands inherent to many physical activities (Best, 2010). The basic assumption of their cognitive stimulation hypothesis, is that coordinatively demanding and non-automated physical activities activate the same brain regions that are used to control higher order cognitive processes (Best, 2010; Pesce, 2012; Tomporowski et al., 2015). For the relation between *acute* physical activity and cognition, it is assumed that these cognitive demands are leading to better cognitive performance by pre-activating the same cognitive processes during physical activity as the ones used in a subsequent cognitive task (Budde et al., 2008). For example, when playing a physical version of the number connexion test (Oswald and Roth, 1987), requiring the subject to discriminate visual stimuli, perform fast mental operations, and react with an appropriate motor response, performance, as a result, is facilitated in a consecutive cognitive test involving the same cognitive processes (Schmidt et al., 2016). However, findings of acute studies explicitly testing this hypothesis remain equivocal, with some revealing positive effects on cognitive performance in favour of the cognitively challenging condition (Budde et al., 2008; Pesce et al., 2009; Jäger et al., 2014; Benzing et al., 2016; Schmidt et al., 2016), some finding no difference (Best, 2012; Jäger et al., 2015) and others even reporting detrimental effects compared to physical activity without cognitive challenges (Gallotta et al., 2012, 2015; Egger et al., 2018).

When it comes to *chronic* physical activity interventions, there is tentative evidence that cognitively engaging aerobic exercise, in which strategic behaviours, complex motor coordination, and adaptation to changing task conditions is required, benefits children’s EF (i.e., working memory, inhibition, and shifting) more than non-engaging simple and repetitive actions (Chang et al., 2013; Schmidt et al., 2015; Koutsandreou et al., 2016; Pesce et al., 2016; Van der Niet et al., 2016). Interestingly, the shift from the quantitative to the qualitative characteristics of physical activity is accompanied by a preference for psychological mechanisms explaining the relationship between physical activity and cognition. Thus, at least in terms of their referred theoretical assumptions, this lastly evolved line of exercise and cognition research is approaching the embodied cognition research, which will be described below.

## Embodied Cognition Research

The roots of embodied cognition research can be placed in literature related to memory of action events (Engelkamp and Zimmer, 1989; Zimmer et al., 2001), suggesting that performing actions leads to the construction of rich and elaborative representations that enhance memory recall. The “enactment effect” engages the motor system, in which encoding is facilitated compared to listening or just observing the same events. The enactment effect sets the foundations for the embodied cognition perspective.

This notion was further developed in psychological research in different domains, such as neuroscience (Martin, 2007), memory (e.g., Glenberg, 1997), and language research (e.g., Glenberg and Kaschak, 2002; Lindemann et al., 2006; Zwaan and Taylor, 2006). It was concluded from this psychological research that the emphasis should be placed on the role of bodily form, real-world action, and environmental influences. In that sense, embodiment includes the actual bodily states and the simulations of the experience linked to the cognitive processes (i.e., action, perception, and introspection; Niedenthal et al., 2005). Based on this tenet, a substantial body of research emerged using gestures during learning (e.g., Alibali and Nathan, 2012).

Expressing information in gestures is one specific type of sensorimotor experience that has been shown to be effective for children’s learning in different domains, such as mathematics (e.g., Cook et al., 2008; Novack et al., 2014), science (e.g., Ping and Goldin-Meadow, 2010; Lozada and Carro, 2016), and language (e.g., Tellier, 2008; Macedonia and Knösche, 2011). Research into the effects of movements in the form of gestures has mainly used learning as outcome variable, and used cognitive explanations to explain these effects.

### Cognitive Explanations for the Effects of Movements on Learning

Building up a conceptual framework, there are several explanations for the cognitive benefits of the human movement effect on learning. In the educational research field, there is a growing body of research that supports the positive effects of engagement in sensorimotor experiences (i.e., the body) during learning activities on learning (e.g., Pouw et al., 2014). Central to this respect, is the theoretical framework of embodied cognition, which holds that cognitive processes are profoundly dependent upon body’s interactions with the world (Wilson, 2002; Barsalou, 2008; Glenberg et al., 2013). Research shows that visual and motor processes in the brain are involved during cognitive tasks, such as text comprehension, mental arithmetic, reasoning, and problem solving, while semantic codes are activated during specific motor actions, illustrating the inter-relatedness of cognitive and sensorimotor processes.

Complementary to the embodied cognition theoretical framework is the evolutionary explanation of cognitive load theory, which categorises information into biologically primary and secondary (Geary, 2008, 2012; Paas and Sweller, 2012): biologically primary knowledge evolves naturally without explicit instruction. For example, the development of native language or the use of movements have been acquired effortlessly and sometimes even unconsciously. Biologically secondary knowledge usually occurs after explicit instruction during formal schooling (e.g., mathematics, science). This type of information can be conveyed through conscious processing, meticulous attention, and effortful deliberate practise. It can be argued that the human motor system, as a form of biologically primary knowledge can be employed to support learning of complex tasks (i.e., biological secondary knowledge).

In addition to the embodied and evolutionary approach, the cognitive explanation suggests that including relevant movements during learning rather than only observing or listening to a task creates a richer memory trace in long-term memory, rendering it more accessible during recall (Feyereisen, 2009; Madan and Singhal, 2012). Mental representations consist of an elaborated network of information regarding concepts. New information is processed in the working memory. When this information is embellished with sensorimotor experiences stemming from multimodal resources (i.e., visual, auditory, and kinaesthetic), it leads to the construction of higher quality mental representations or cognitive schemas, and in turn faster and better memory performance (Goldin-Meadow et al., 2001; Ping and Goldin-Meadow, 2010; Cook et al., 2012). Schema enrichment for students’ engagement in action along with perceptual tasks can also be linked to the dual coding theory (Clark and Paivio, 1991). This theory emphasizes that students who are involved with motion and perception are able to connect the verbal and visual cues with their kinaesthetic “imagery” (Clark and Paivio, 1991). For instance, a mental image for the word “Bunsen burner” comprises a visual image of the object, auditory, and olfactory images for the sound and smell of gas, and motor images for adjusting the flow of gas (Clark and Paivio, 1991). Overall, using tactile and kinaesthetic cues can offload some of the working-memory resources, as information is processed simultaneously but not through the same system (for a review see, Risko and Gilbert, 2016). Studies in the domains of mathematics (Goldin-Meadow et al., 2001) and Piagetian conservation tasks (Ping and Goldin-Meadow, 2010) have shown that hand gesturing can reduce a speaker’s cognitive load during instruction and problem solving.

Finally, in accordance with the mirror neuron system, sensorimotor actions, thoughts, or words activate mirror neurons, and in turn mental representations responsible for movements, such as action and language for example that are both located next to the Broca’s area in the brain (Aziz-Zadeh et al., 2006; Aziz-Zadeh and Damasio, 2008). The mental imagery of the movement, supplementary to the physical execution of the movement, is an alternative way which can possibly achieve the same gains in cognitive learning. The mirror neuron system has been linked with action understanding as well as the ability to observe and imitate others’ actions (Rizzolatti and Craighero, 2004). However, this notion has been under great dispute (Caramazza et al., 2014; Hickok, 2014). Van Gog et al. (2009); see also Paas and Sweller, 2012) have argued that the mirroring capacity of the human brain can be used in learning when a motor component is encompassed in the cognitive tasks. Motor imagery could be combined with a concurrent demonstration of the same action, enforcing the involvement of the mirror neuron system, to produce more effective motor learning outcomes (Eaves et al., 2016). However, more research is needed to shed light on this notion.

Nevertheless, solid empirical evidence attests various concrete examples of the aforementioned explanations for the benefits of embodiment on learning. Most of the existing research has focused on subtle movements, such as gestures, with fewer examples existing of research looking at whole-body movements. These movements do not provoke any physical exhaustion, but they are a significant adjunct of the learning process. Gestures can be distinguished into beats (non-representational gestures), iconic (implying a perceptual relationship between concrete concepts), metaphorical (having a narrative character for abstract concepts), and deictic gestures (when the speaker points to actual objects; Roth and Lawless, 2002; Hostetter, 2011). An illustrative example of a gesture study using iconic gestures, is a study by Cook et al. (2008). They reported that children who were instructed to gesture meaningfully (i.e., children first place their hand on the left side of the equation, then they pose, and finally put their hands under the right side), while practising solving mathematics problems, increased their learning relative to children who were told to only speak during the practise phase. These results provide support for a causal role for gesturing in learning. Moreover, Lajevardi et al. (2017) showed that mimicking gestures were beneficial for learning to write foreign language characters, both in instructional designs, including animations and static graphics. Although the exact mechanism is not completely understood, these findings are in line with the embodied cognition hypothesis, that expressing information in multiple modalities can promote the construction of higher quality cognitive schemas more than conveying the same information in speech alone.

Another demonstrations of the benefits of embodiment on learning comes from a study by Goldin-Meadow et al. (2001) who explored how gesturing during one task (i.e., explaining solution to math problems) impacted the performance on another task (i.e., remembering letters/words). Both children and adults who were given the dual task of remembering letters (for adults) or words (for children) while explaining a difficult math problem remembered more items when gesturing than when not gesturing (Goldin-Meadow et al., 2001). The higher score on the secondary task after explaining the math problem solution with gestures indicates that gesturing reduced the cognitive load imposed by math explanation freeing up resources that can be used on a secondary memory task. It is important to note that only task-relevant gestures, defined as movements that are in coordination with the content of the speech, reduce the cognitive load of explanation (Cook et al., 2012). Further evidence for the relation between gesturing and cognitive load is provided by studies that found a positive relation between cognitive task complexity and gesture frequency, and studies that found gesturing to be especially beneficial for individuals with low working memory capacity (Chu and Kita, 2011; Pouw et al., 2016).

Finally, some technology-based educational studies have not only used gestures, but also whole-body movements to enhance learning. These studies are based on the theory that knowledge is grounded in body-based actions, where the body works as a sensorimotor metaphor, converting the abstract concepts into tangible examples (e.g., Johnson-Glenberg et al., 2014). For example, in a recent study, a group of middle school students were put in the role of an asteroid. By using their bodies in an immersive, whole-body, interactive environment they learned about planetary motion and gravitational forces (Lindgren and Johnson-Glenberg, 2013). Another group of students learned about planetary motion using a desktop version of the same simulation. Results of the study showed that the group of students in the interactive digital environment having an embodied learning experience had significantly higher learning performance, higher levels of engagement, and more positive attitudes toward science than the other group using a desktop computer.

## A Matrix From the Prism of Task Relevance and Integration Levels

Although this is a narrative review, given the very broad inclusion of (1) physical activity tasks, (2) cognitive/learning outcomes, (3) length of interventions and of single activity bouts, and (4) intervention settings, arising from the two research traditions (i.e., exercise and cognition research and embodied cognition research), a minimum inclusion criteria was applied: acute and chronic physical activity interventions, gross and fine-motor training tasks, and age (children, adolescents, and adults).

**FIGURE 1:**
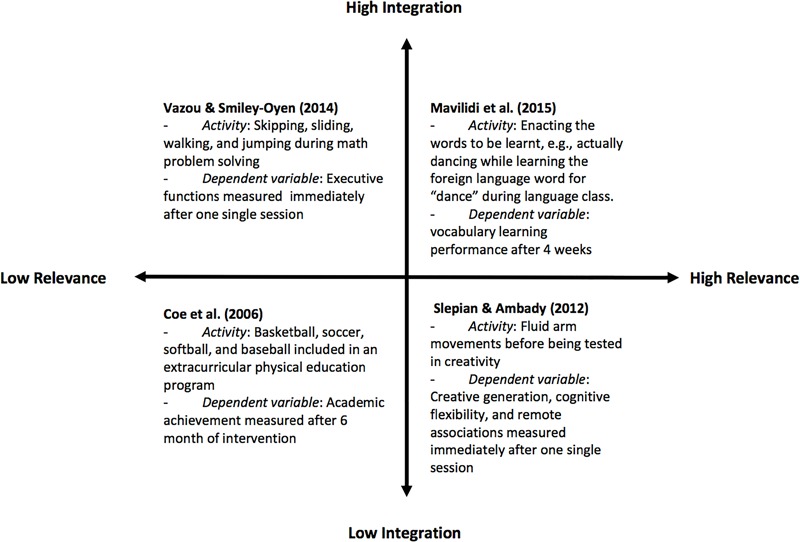
A cognitive-motor matrix displaying a coarse relative comparison of a select number of studies across two dimensions: the horizontal dimension reflects the level of relevance between the physical and the cognitive and learning task and the vertical dimension reflects the level of integration of the physical task and the cognitive or learning task.

The second part of this review will categorise the selected studies on a continuum based on the relevance of the movements for the cognitive/learning task and the integration of the movements into the cognitive/learning task (see Figure 1):

1. Relevance of the movement for the cognitive/learning task: this categorisation refers to the level of embodiment or relatedness of the physical with the cognitive task. Of a dominant role to this continuum is the factor of embodiment, referring to the enactment of concepts using the body (Lindgren and Johnson-Glenberg, 2013). These range from no embodiment, where the movements are not related with the cognitive tasks, to high embodiment, in which whole-body movements are engaged, meaningfully related with the learning tasks. For example, performing locomotor skills such as skipping, sliding, walking, and jumping, while avoiding obstacles when working on math problems can be considered as non-task-relevant movements (Vazou and Smiley-Oyen, 2014), whereas dance movements that children perform when learning the foreign language word for dance (Mavilidi et al., 2015) can be considered as task-relevant movements. Both activities, however, are integrated into the corresponding cognitive/learning task.
2. Integration of the movement into the cognitive/learning task: this categorisation refers to the temporal connexion of the movements with the learning task. If the movements are performed before or after the learning task with an interval in between, the integration is low. If movements are performed during the learning task, the integration is high. For example, performing fluid arm movements immediately before being tested in creativity, can be considered as being task relevant, but it is not integrated into the cognitive/learning task (Slepian and Ambady, 2012). At the other end, an additional after-school physical education programme has low integration level, as the physical activities occurred beyond the academic instruction time and are considered as having low task relevance (Coe et al., 2006). A continuum exists ranging from non-integrated movements, where there is no temporal overlap between movements and the learning task, to integrated movements, where the movements are connected and included during learning.

Figure 1 plots four examples chosen for each quadrant of the 2 × 2 matrix based on their relevance and integration with the cognitive/learning task. Research on exercise and cognition (i.e., physiological mechanisms) falls in the low-relevant quadrants, whereas research on integrated movement studies (i.e., embodied learning) falls into the high-relevant quadrants. A discussion of the studies belonging to each of the quadrant is presented below.

### Bottom Left Quadrant: Body Movements Not Relevant for, and Not Integrated Into the Cognitive/Learning Task

Research of this area involves studies examining the acute effects of exercise on cognition (Hillman et al., 2009), activity break studies, and afterschool programmes focusing on physical fitness and its indirect relationship with cognitive and academic performance (Castelli et al., 2011; Davis et al., 2011), Starting with the acute exercise studies, research has suggested that a single bout of exercise improves cognitive performance on attention demanding tasks in preadolescent children. For instance, Hillman et al. (2009) found that single, acute bouts of moderate treadmill walking improved the cognitive control of attention in children as measured with a modified flanker task. Applied aspects of cognition involved in school-based academic performance improved from this single bout of exercise also. In another similar study, preadolescent children performed better on cognitive tasks measuring attention and inhibitory control after short bouts of 20-min walking compared with children who remained seated (either walking or seating breaks were incorporated in between the cognitive tasks for three sessions; Drollette et al., 2012).

Also in the classroom itself, non-integrated and non-relevant activity can take place. Activity breaks, for example, are interspersed between phases of learning, but they do not overlap in time with the learning tasks in the classroom. Several studies investigated forms of physical activity that occurred in the classroom aside from gym classes, recess, or breaks (Katz et al., 2010; Telford et al., 2012; Howie et al., 2015). Generally, these studies investigated the effects of short physical activity breaks (most commonly aerobic routines for 5–20 min), or ways to incorporating physical activity into the learning activity that were either intended to increase learning through motor actions, or to provide purely an exercise moment for children to increase energy expenditure. The studies examined whether and how introducing activity breaks in a classroom environment, impacted health, cognitive skills (Hill et al., 2010), attitudes (mood, motivation; Vazou et al., 2012), academic behaviours (i.e., on-task behaviour, concentration, motivation; Greico et al., 2009; Webster E. K. et al., 2015), and academic achievement (i.e., reading literacy scores or math fluency scores; Katz et al., 2010) of children. In general, reviews on the relationship between activity breaks with aspects of academic performance show that activity breaks either have positive effects or do not adversely impact cognitive function and academic performance (Centers for Disease Control and Prevention, 2010). For example, research revealed that 4 min of high-intensity interval activity in class enhances selective attention in children aged 9–11 years (Ma et al., 2014). Likewise, running games or performing fundamental movement skills such as hopping, skipping, and jumping in the classroom could enhance fluid intelligence and academic achievement in 9–11 years old children (Reed et al., 2010).

Finally, afterschool physical activity programmes fall under the category non-integrated, non-relevant physical activity. For example, the 9-month afterschool physical activity programme “FITkids” for children 7–9 years old aiming to ameliorate brain health and cognitive performance found improvements in heart rate as a measurement of physical activity, physical fitness, inhibition, and cognitive flexibility in children who participated in the afterschool intervention group, as compared to the control group (Castelli et al., 2011; Hillman et al., 2014). The physical activity intervention offered two hours of physical activity each day after school, in which children were requested to participate for up to 40 min.

In the study of Coe et al. (2006), students were enrolled into a physical education programme (including activities such as basketball, soccer, softball, and baseball), or an extra instruction class either in the first or second semester for 55 min every day. Their academic achievement was measured by students’ grades on four core courses (mathematics, science, English, and world studies), and a standardised test. Academic achievement was not altered by the moderate intensity in the physical education class; however, children who partly or fully met the guidelines of 30 min moderate activity per day for at least 5 days per week, or 20 min of vigorous activity per day for at least 3 days per week, had higher grades than students who were not engaged in vigorous physical activity in both semesters.

Davis et al. (2011) assessed 7- to 11-year old children’s EF after randomly assigning them to a 40 or 20 min/day exercise programme, or a no exercise control condition for 3 months. Children in the high dose exercise group completed two 20-min bouts per day, whereas children in the low dose group completed a 20-min bout of exercise and 20-min sedentary activities (e.g., drawing, board and card games). Standardised cognitive assessments, achievement measures, and fMRI were used in the study. Results demonstrated that regular aerobic exercise advanced cognitive functioning in both exercise groups compared to the control group, but with more pervasive effects observed in the high dose exercise group. The high dose exercise group also outperformed the other groups in math scores.

### Top Left Quadrant: Body Movements Not Relevant for, but Integrated Into the Cognitive/Learning Task

These studies used active workstations (e.g., standing and treadmill desks, cycling workstations) while completing cognitive tasks, physical activity learning games, and exergaming in the classroom. The first type of integrated, but non-relevant, physical activity can be identified in a new area of research focusing on the effects of active workstations on cognition. Several meta-reviews report a positive relationship between active workstations, mostly examined in office environments, thereby resulting in reductions in sitting time for adults, increments in energy expenditure, and improved health (Torbeyns et al., 2014; MacEwen et al., 2015). Notwithstanding the health benefits, research into the cognitive effects and computer task performance when using these active workstations remains limited. Further investigation is needed before conclusions can be drawn. Research to-date seems to indicate that low-intensity exercise does not compromise cognitive functions (e.g., Ruiter et al., 2017). For example, Pilcher and Baker (2016) assessed undergraduate students’ performance, motivation (self-reported answers regarding enthusiasm, energy, drive, eagerness, and morale), and engagement (self-reported responses regarding subjective performance, attention, and absorption), when they used a stationary desk bike with a desk top and a traditional desk with chair during the completion of cognitive tasks. Although cognitive performance did not differ, cycling desks improved positive affect, motivation, and morale. By contrast, studies of Schmidt-Kassow and colleagues showed that treadmill walking (Schmidt-Kassow et al., 2014) and cycling (Schmidt-Kassow et al., 2010) during vocabulary encoding improved subsequent recall. The authors attributed this improved performance to the temporal alignment of stimulus presentation and motor activity.

Since active workstations are a novelty, particularly within a school setting, there is currently only one systematic review that has investigated the physiological effects of standing desks interventions within the classroom setting (Sherry et al., 2016), suggesting that energy expenditure is increased when using standing desks within the classroom. Overall, implementing active workstations in classrooms could be used to decrease sedentary behaviour with no detriment of cognitive performance; however, more research is needed to glean insight in active workstations’ potential as cognitive enhancers.

Other types of classroom-based physical activity can also be gathered under the category of non-relevant but integrated movements. For example, Vazou and Smiley-Oyen (2014) integrated a 10-min bout of acute aerobic exercise into math lessons. In the integrated condition, preadolescent children performed locomotor skills such as skipping, sliding, walking, hopping, leaping, bear and crab walking, and jumping to avoid obstacles when working on math problems. For example, when the answer was an odd number, students were crab walking, otherwise in case of an even number, they were bear walking. In the seated math practise condition, no bodily movements were involved. They found that elementary children in the integrated physical activity group showed significant improvements on accuracy while performing a cognitive task for inhibitory control, and higher scores on enjoyment compared with the seated math practise group. The authors concluded that classroom-based physical activity are an enjoyable and realistic strategy to increase physical activity and facilitate EF in children.

Finally, an exercise and game-based learning approach linking digital technology with exercise and learning, also known as exergaming, can be placed in this quadrant. Exergaming is a developing area of research with yet to be established results. It seems a promising way to increase physical activity, improve general coordination skills, motivation, and cognitive outcomes (e.g., improved attention and visual–spatial skills) through linking exercise with digital technology and learning (Staiano and Calvert, 2011).

Exergaming has also been implemented in elementary school for different learning contents such as math, history, and languages, and with varying difficulty levels (Lucht and Heidig, 2013). Higher scores in cued recall were found in the experimental condition, in which children had to jump and move as quickly as possible on a sensor pad when playing a vocabulary game compared to the traditional sedentary condition. The integration of physical activity into learning games seems to be enjoyable and engaging, holding promise for learning in children. It should also be noted that these studies had high ecological validity as they took place in real-life situations and existing school lessons.

To conclude, it is premature to infer about the effectiveness of active video games on augmenting energy expenditure and learning; however, active video games may have an additional benefit on engaging players with light-to-moderate physical activity compared to other sedentary behaviours such as passive video games and rest (LeBlanc et al., 2013). Given the length of time and relatively high-frequency that children and adolescents spend on video gaming (at least 1 h per day on the weekdays and 1.5 h on the weekend for children 10–19 years; Cummings and Vandewater, 2007), successfully designed exergaming is a promising method to positively impact physical activity levels and cognition in children.

### Bottom Right Quadrant: Body Movements Relevant for, but Not Integrated Into the Cognitive/Learning Task

This kind of research can both be found in the embodied cognition and in the exercise and cognition research tradition. Whereas the studies from the embodied cognition literature involve effects of bodily movements on several measures of cognition such as insight problems or creativity (mostly arm movements at a very basic physical activity level), studies out of the exercise and cognition literature predominantly investigate the effects of whole-body movements on cognitive measures, such as EF. The movements occurred prior or after the cognitive task, with the key concept being the accordance between the movement and the cognitive task. Thus, these movements function as simulated actions, indirectly fostering information processing (McMorris et al., 2009; Alibali and Kita, 2010). It is argued that the body can work as a scaffold, or conceptual metaphor, to abstract cognitive contents (Williams et al., 2009; Landau et al., 2010), guiding higher order cognitive processing (Thomas and Lleras, 2009).

Thomas and Lleras (2009) allocated university students to exercise breaks either consistent with (swing group) or inconsistent with (stretch group) an insight problem that they had to solve immediately afterward. They had to solve Maier’s two-string problem, which they could only solve by swinging one of the ties with an object attached. The experiment consisted of eight attempt intervals with 20-s exercise and 100-s problem-solving periods. It was found that the swing group was better in solving the two-string problem than the stretch group, without being aware of the swinging arm movements as overt hints to problem-solving. Consistent with these results, Werner and Raab (2013) found a movement-specific influence on participants’ solution of two-string and water-jam problems. They assigned adult participants to two movement groups that were congruent with the problem solutions or a control group. To this end, Raab and Green (2005) suggested that movement goals rather than the arm position cause activation and internal evaluation process, affecting performance in a word association task.

Another study compared effects of fluid with non-fluid movements on creative thinking (Slepian and Ambady, 2012). A set of drawings was designed in which undergraduate students had to trace either arm movements with or without line curvature (fluid and non-fluid movements, respectively). Subsequently, participants were assessed in creative generation, cognitive flexibility, and remote associations. Fluid movement enhanced creativity in all three domains, even though participants did not consciously perceive this positive affect.

Glenberg et al. (2008) provided evidence on the causal link between language comprehension and the motor system. Participants were required to perform a 20-min repetitive transfer motor task in which they had to move 600 beans from a wide-mouthed container to a narrow-mouthed container. After moving the beans, they had to read sensible and non-sensible sentences describing transfer events with abstract (e.g., “responsibilities”) and concrete (e.g., “cards”) objects toward or away from themselves. It was found that the participants’ concrete and abstract language comprehension differed based on the motor task (i.e., arm movements toward vs. away from the body, and right-hand vs. left-hand index finger).

A last series of studies in which the adopted movements can be considered as being relevant for, but not integrated into the cognitive task, are the ones searching for identical elements (Woodworth and Thorndike, 1901) between the qualitative characteristics of specific physical activities and the cognitive task used to measure the dependent variable, such as attention or EF (Jäger et al., 2014, 2015; Benzing et al., 2016; Schmidt et al., 2015, 2016; Egger et al., 2018). For example, in a group randomised controlled trial, Schmidt et al. (2015) designed a 6-week intervention in physical education enriching traditional team games, e.g. basketball, with additional instructions in which the children had to keep in mind different rules (updating), react appropriately to acoustic cues by inhibit pre-potent movements (inhibition), as well as switch between different situations and rules (shifting). At the end, they compared this cognitively challenging intervention to an aerobic as well as an active control condition, measuring their core EF (updating, inhibition, and shifting). Despite similar benefits in aerobic fitness of the children of both the aerobic and the enriched condition, only the children of the enriched condition displayed higher gains in shifting performance.

### Top Right Quadrant: Body Movements Relevant for, and Integrated Into the Cognitive/Learning Task

The studies included almost exclusively address years of children’s development in the school context. First, subtle movements, mainly gestures, are described to continue with studies involving whole-body movements, and finally gross motor movements in the form of physical activity.

#### Subtle Movements – Gestures

Well known in this field are the mathematical studies in which 9- to 10-year-old children were taught to produce an abstract gesture as a tool for solving new mathematical-equivalence problems in the form of 5 + 2 + 4 = __ + 4 (Goldin-Meadow et al., 2001, 2009). In solving these problems, children first had to produce a V-point gesture to the first two numbers to represent addends of an equation, and then point at the blank were the sum of the equation should be put. Children who were instructed to make these gestures during a math lesson benefited more from math instruction than children who were not prompted to make these hand movements. They retained more of the knowledge and were able to extract the underlying grouping strategy, although they were never explicitly told what the gestures stood for.

Gestures as a form of indexing and enactment have also been shown to facilitate learning in domains of language comprehension, and primary and secondary language acquisition (Kelly et al., 2010; Macedonia et al., 2011). For example, in case of vocabulary learning, Macedonia and Klimesch (2014) taught undergraduate students novel words from an artificial language. Participants enrolled in the gestural condition in which they had to read, repeat the words they heard, and imitate the researcher who was enacting the words with symbolic gestures, were able to recall more words and for a longer period of time than those who had just read, heard, and repeated the same words. Similarly, gestures can be used to enact sentences or storeys to improve reading comprehension: the process of reading a text and understanding its meaning (Fischer and Zwaan, 2008; De Koning and van der Schoot, 2013). For example, Glenberg et al. (2004) found that children who were manipulating (actual and imaginative) toys referred to in the text they were reading (e.g., farm animals) remembered more action sentences from the storey and showed a better comprehension of the text than children who only read the storey. In another recent study on reading comprehension, Berenhaus et al. (2015) compared the effect of indexing (i.e., mapping words to objects) and enactment on memory of a narrative. Children in the indexing condition used play mobile figures to perform the storey while children in the enactment condition took on the role of the characters and acted out the storey with gestures and emotional expressions. The results revealed that children in the enactment condition remembered more descriptive parts of the storey than children in the read only condition. Moreover, both enactment and indexing benefited children with poor reading ability. Finally, iconic gesturing in the form of actions or object-manipulations has been proven useful for learning, facilitating problem-solving and vocabulary retrieval by activating relevant perceptual-motor information (e.g., Macedonia et al., 2011).

#### Whole-Body Movements

In the area of math, it has been shown that whole-body movements can help children adequately develop a spatial representation of number magnitude. For example, Ruiter et al. (2015) examined the process of number building (two-digit numbers) in first-grade children by making steps on a ruler across the floor. In the two-movement condition, children made small, medium, and large steps representing different number units of 1, 5, and 10, respectively, whereas in the two control conditions, children had to verbally construct the two-digits numbers. Results showed higher test performance when children were engaged in full-body movements.

Shoval (2011) examined the effects of cooperative active learning when learning angles in geometry class. Second- and third-grade students were enrolled either in an experimental or a control condition. In the experimental condition, children collectively formed a circle with their bodies to learn about the circle, whereas children in the control condition learned about the subject geometry through the sedentary conventional method. It was found that the experimental group using movements in a cooperative learning setting reached better results than the conventionally taught group without movements.

Finally, a recent study looked at the effects of whole-body movements and gestures on learning foreign language vocabulary in 4-year-old children (Toumpaniari et al., 2015). First, children were shown flashcards with animal names. Children were assigned to one of three groups in which they had to recall animal words (a) through performing physical activities and gestures relevant to the animal words to be learned, or (b) gesturing related to the animal words, or (c) through the conventional way. Results showed that learning a foreign language vocabulary through physical activities and gestures was considered the most enjoyable way of learning, and resulted in the highest learning outcomes. In this study, no physiological outcomes were measured.

#### Blended Approach: Combining Gross Motor Movements With Learning

Studies consisting of whole-body movements in the form of classroom-based physical activity are presented, measuring both cognitive and physical activity outcomes. For instance, during the daily 15-min of classroom-based physical activity, elementary school children could learn geometry, by forming different shapes with their bodies (e.g., squares or triangles) while walking or hopping on an outdoor playfield, geography, by running to the appropriate area allocated for one of the cardinal directions, or spelling, and by hopping onto a floor mat with alphabet letters onto it (Donnelly and Lambourne, 2011). The results revealed significant improvements in academic achievement as well as a significantly lower increase in body mass index among children in the experimental compared to the control classrooms.

A series of intervention studies integrating physical activity into learning tasks lasting 10–15 min per day also demonstrated prominent effects in early childhood (Mavilidi et al., 2015, 2016, 2017, 2018). Children were enrolled in the integrated condition in which children were engaged in meaningful, task-relevant physical activities (e.g., dancing while learning the word dance; imitating animal movements relevant to animals living in each continent while learning about the continents and animals living there; moving from the Sun to Mercury and repeat the same process for all planets while learning about the planets’ names and their distance from the Sun, counting numbers while walking on foam blocks of numbers), a condition involving task non-relevant physical activities (e.g., running around the room before the learning task), or control condition (sedentary way of learning). Overall, the integrated condition had higher learning scores, was more physically active and enjoyed learning the most, compared to the control condition.

## Discussion

### Converging Different Lines of Research Toward a Blended Approach

This review summarised theoretical and empirical evidence, connecting action with perception, cognition, and learning. Previous research has revealed the positive association of physical activity and mental health (White et al., 2017), as well as the synergistic effects of physical activity, and fitness on cognition and academic performance (Lees and Hopkin, 2013). Concomitantly, research about embodiment evaluates the role of the body (e.g., gestures, object manipulation) during the learning process, arguing that embodying knowledge through task-relevant movements can positively impact learning (Barsalou, 2008). Overall, a new view of embodied cognition can be supported in this review, in which motor and cognitive control, are related to the same cognitive processes (Pesce and Ben-Soussan, 2016).

Summarizing the cognitive explanations for including movements during learning suggested in this work, the benefits lie in their associative activation of imagery. Imagery is one way to strengthen the motor representation of the task, which can improve motor performance, and may presumably have a potential benefit on learning. In fact, mental imagery (consisting of visual, motor, or kinaesthetic imagery, as related to our senses) has been linked to better motor performance, general exercise experience, and a variety of mental skills (such as anxiety management, confidence, and concentration; Short et al., 2001; Weinberg, 2008). Motor imagery ability may be a key function in determining the extent to which a learner may prepare, rehearse, and subsequently “embody” the activity, long after the physical execution of the task itself. Jeannerod (2001) suggests that the motor representation produced can be achieved via action observation, and can be functionally equivalent to motor imagery. To this end, a study in adult participants using fMRI explored the cerebral structures engaged in visual and kinaesthetic imagery, concluding that overlapping similar activations (i.e., in motor-related regions and superior and inferior parietal lobules) occurred during physical executing the task, and both visual and kinaesthetic imagery, in comparison with the perceptual condition (Guillot et al., 2009). However, different brain patterns were activated during visual and kinaesthetic imagery, with kinaesthetic imagery engaging more motor-associated structures. A study in 120 school children (9–10 years) examined whether participation in physical activity and movement imagery ability can predict active play imagery (Guerrero and Munroe-Chandler, 2018). It was found that active play imagery can be determined by age, participation in physical activity, and ability to use external visual imagery.

On the other hand, examining the physiological mechanisms of movements on cognition and learning performance, research in physical activity, exercise, and fitness, vary vastly in intensity, duration of bouts or interventions, cognitive challenges, and time relation between the physical and cognitive engagement. Several types of physical activity interventions such as enhanced or enriched physical education, classroom-based physical activity, activity breaks or active play during recess, extracurricular physical activity interventions, or after-school programmes were included in this review. The contextual factors of physical activity inclusion (e.g., place, type, and duration) seem to be determinant when inferring about the association among physical activity and cognition (i.e., EFs such as working memory, inhibition, and cognitive flexibility, metacognitive functions such as abstract reasoning, problem-solving, and cognitive life skills such as self-regulation, goal setting), academic achievement (i.e., mathematics, language scores), and academic behaviours (i.e., on-task behaviour; Álvarez-Bueno et al., 2017).

Physical activity so far revealed a potential to elicit improvements in cognitive performance. However, there are substantial differences concerning the effects of effortful and prolonged bouts of physical activity on cognition, and those of cognitively engaging movements of low intensity and duration. For example, negligible effects of low-intensity and short physical activity tasks are reported in reviews on acute exercise and cognition (Tomporowski, 2003; Chang et al., 2012; Vazou et al., 2016). By contrast, more pronounced effects can be observed during cognitively engaging physical tasks (meta-analysis of Vazou et al., 2016). The mentally enriched and cognitively engaging physical activities offer a range of different inherent qualitative characteristics (i.e., task complexity, novelty, and diversity/variety, emotional activation, selection of suitable mental strategies). These characteristics act as brain stimulators contributing to enhancements in children’s executive functioning (Diamond and Ling, 2016; Vazou et al., 2016). Thus, it can be speculated that if these qualitative characteristics are connected with the learning material, the highest learning effects can be expected.

**FIGURE 2:**
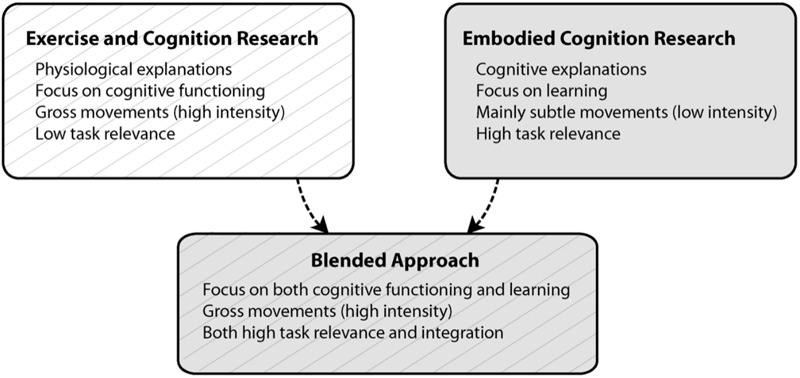
Conceptual framework of the blended approach.

Converging the cognitive and physiological mechanisms of movements, we propose an innovative instructional method that combines task-relevant physical activities integrated with learning tasks (Figure 2). We believe that by adopting this approach, children could benefit from the combined physiological (e.g., increased arousal, neurological alterations in the brain; Best, 2010, 2012) and cognitive effects (e.g., embodied learning; Lindgren and Johnson-Glenberg, 2013) on cognition and learning. Gathering support toward the integrated task-relevant physical activity programmes, they may offer paramount health and cognitive outcomes (meta-analysis of Fedewa and Ahn, 2011; systematic review of Lubans et al., 2016; systematic review and meta-analysis of Morgan et al., 2013; systematic review and meta-analysis of Owen et al., 2016). Existing programmes have already received positive social support and feedback within the school environment, because their versatility takes into account time and budget constraints, and limitations in teachers’ experience (Webster C. A. et al., 2015). Integrated physical activities offer different possibilities and variations based on the level of task complexity, children’s age group, and syllabus’ restrictions. This flexibility promotes high ecological validity including a universal applicability to all classrooms settings in which students are engaged in high quality learning activities in an engaging, motivating, and amusing way. Hence, it is suggested that the classroom instruction integrated with physical activities does not have a detrimental effect on academic time (Sallis et al., 1997; Ward et al., 2006), but rather positions academic content centrally, empowering it.

Notwithstanding, valuable efforts have been taken to increase physical activity through stealth interventions (Robinson, 2010), mostly in school years. Nevertheless, effective programmes in the early years can improve children’s EF (Diamond et al., 2007). Coupling physical with cognitive tasks fosters cognitive development, motivation, and engagement (Leppo et al., 2000; Moreau, 2015). Overall, the integration of movement experiences to learning areas results in children being more physically active, enthusiastic, and attentive to learning tasks, such as math or language (Trost et al., 2008; Mavilidi et al., 2016, 2017, 2018; Riley et al., 2017). We argue that similar efforts should target all age groups in a way that time spent in schools contributes to children’s physical and mental wellbeing, cognitive and socio-emotional functioning, and the development of healthy lifestyles, with ample directions toward prevention of later cognitive delays and building of school readiness (Anderson et al., 2003; Pate et al., 2006; Erwin et al., 2013).

## Conclusion

### Blending Physical Activities With Classroom Instruction

In this review, we have provided an overview of studies dealing with the effects of physical activity on cognition and learning. We identified two different research traditions, which have evolved unconnected and side by side, based on completely different theoretical backgrounds: Exercise and cognition research and embodied cognition research. In categorising the studies with respect to the *relevance for* and the *integration into* the cognitive/learning task, it became obvious that in these two lines of research various methodological approaches are used. Whereas acute exercise and cognition research focuses on the immediate impact of gross motor physical activities on basic cognitive processes, such as attention or EFs, for example, embodied cognition research is more interested in affecting the learning process itself by fine motor movements, such as gestures.

Although considering research into the relationship between physical activity, cognition, and learning through the lens of relevance and integration provides a rough, yet further to be developed categorisation, it offers some insight in how both lines of research could profit from each other. For example, embodied cognition research conducted with children in the educational setting revealed that task-relevant whole-body movements can facilitate foreign vocabulary learning (Mavilidi et al., 2015). However, in these studies, attentional performance immediately after single learning sessions has not been considered. For the educational setting, e.g., lesson scheduling, it could be interesting to know if children’s attention is enhanced or deteriorated after such interventions. On the other hand, exercise and cognition research is so focused on constructs such as attention and EF that, to the best of our knowledge, there are few studies testing whether the effects on these variables also impact learning as a consequence. Therefore, for both lines of research, there might be an added value in considering each other’s theoretical and methodological approaches. In education, physical activity and cognitive activity are typically treated as unrelated processes. By contrast, a more integrated approach is recommended for most effective health and learning outcomes. The current review in no way exhausts the existing literature but rather uses selective examples to draw conclusions and suggest a new instructional method in which physical activities are intermingled with classroom instruction. Results of recent studies confirm that integrated, task-relevant physical activities have paramount effects on learning. Future research is needed to shed light on the required frequency and duration of the classroom-based physical activity programmes, taking into account different age and target groups (including minority or low socioeconomic status children, children of typical development, and diagnosed with developmental disorders), and the feasibility of their implementation in “real-world” settings (including teachers’ preferences, sustainable resources, construction of “user-friendly” manuals, and guidelines for teachers).

## Author Contributions

MM and MR equally contributed to the completion of the manuscript and the development of the original concept (shared first authorship). MS, SL, AO, PC, and FP contributed to the development of the original concept, supervised drafting of the manuscript, and reviewed it for important intellectual content.

## Conflict of Interest Statement

The authors declare that the research was conducted in the absence of any commercial or financial relationships that could be construed as a potential conflict of interest.
